# Effects of bright light treatment on psychomotor speed in athletes

**DOI:** 10.3389/fphys.2014.00184

**Published:** 2014-05-12

**Authors:** Mikko P. Tulppo, Heidi Jurvelin, Eka Roivainen, Juuso Nissilä, Arto J. Hautala, Antti M. Kiviniemi, Vesa J. Kiviniemi, Timo Takala

**Affiliations:** ^1^Department of Exercise and Medical PhysiologyVerve, Oulu, Finland; ^2^Department of General Practice, Institute of Health Sciences, University of OuluOulu, Finland; ^3^Department of Biology, University of OuluOulu, Finland; ^4^Department of Diagnostic Radiology, University of OuluOulu, Finland; ^5^Department of Sports and Exercise Medicine, Oulu Deaconess InstituteOulu, Finland

**Keywords:** transcranial treatment, seasonal darkness, cognition, motor speed, ice-hockey

## Abstract

**Purpose:** A recent study suggests that transcranial brain targeted light treatment via ear canals may have physiological effects on brain function studied by functional magnetic resonance imaging (fMRI) techniques in humans. We tested the hypothesis that bright light treatment could improve psychomotor speed in professional ice hockey players.

**Methods:** Psychomotor speed tests with audio and visual warning signals were administered to a Finnish National Ice Hockey League team before and after 24 days of transcranial bright light or sham treatment. The treatments were given during seasonal darkness in the Oulu region (latitude 65 degrees north) when the strain on the players was also very high (10 matches during 24 days). A daily 12-min dose of bright light or sham (*n* = 11 for both) treatment was given every morning between 8 and 12 am at home with a transcranial bright light device. Mean reaction time and motor time were analyzed separately for both psychomotor tests. Analysis of variance for repeated measures adjusted for age was performed.

**Results:** Time × group interaction for motor time with a visual warning signal was *p* = 0.024 after adjustment for age. In Bonferroni *post-hoc* analysis, motor time with a visual warning signal decreased in the bright light treatment group from 127 ± 43 to 94 ± 26 ms (*p* = 0.024) but did not change significantly in the sham group 121 ± 23 vs. 110 ± 32 ms (*p* = 0.308). Reaction time with a visual signal did not change in either group. Reaction or motor time with an audio warning signal did not change in either the treatment or sham group.

**Conclusion:** Psychomotor speed, particularly motor time with a visual warning signal, improves after transcranial bright light treatment in professional ice-hockey players during the competition season in the dark time of the year.

## Introduction

Bright light treatment has various positive psychophysiological effects. An acute improvement of cognitive performance in healthy subjects at night (Campbell and Dawson, 1990; Badia et al., 1991; Daurat et al., 1993; Cajochen et al., 2000; Lockley et al., 2006) and during the day (Phipps-Nelson et al., 2003; Ruger et al., 2006). Recently, Chellappa et al. showed that cognitive performance, and particularly reaction time, in healthy young men is improved acutely by blue-enriched bright light treatment (Chellappa et al., 2011).

Timonen et al. raised the question of whether non-visual effects of light (like improved reaction time) could be partly mediated via a non-retinal pathway (Timonen et al., 2012). Ten out of 13 patients suffering from seasonal affective disorder (SAD) achieved full remission after 4 weeks of transcranial brain-targeted bright light treatment via the ear canals (Timonen et al., 2012). A significant amount of light does penetrate the skull bone and reaches the brain in mammals (Ganong et al., 1963). Very recently it has been suggested that a new mechanism of direct, non-visual photoreactivity of the brain occurs via several naturally expressed, inborn opsin genes which have been found to express in mRNA and protein levels in mouse and human brains (Blackshaw and Snyder, 1999; Kojima et al., 2011; Nissilä et al., 2012). As evidence of brain reactivity to non-visual light, Starck et al. showed that immediate transcranial bright light treatment increased resting state brain activity of secondary visual and sensorimotor networks when compared with sham treatment in healthy subjects studied by means of functional magnetic resonance imaging (fMRI) (Starck et al., 2012). Intriguingly, the same visual and sensorimotor networks show altered resting state activity in subjects suffering from winter-type SAD, triggered annually by darkness (Abou Elseoud et al., 2014).

Psychomotor performance is an important determinant of performance in sports, specifically in those requiring fast decision making and execution skills. Also, particularly in professional sports, high strain due to the extremely busy competition schedule may result in decreased cognitive performance as an early marker of overreaching (Nederhof et al., 2006, 2007; Hynynen et al., 2008). Overreaching is a condition when “immediately after the period of overload training performance will usually be impaired” and recovery to the normal performance level last days to weeks (Nederhof et al., 2007). The suggested marker of overreaching is psychomotor slowness, measured with motor time and reaction time tests (Nederhof et al., 2006, 2007). High physiological and psychological strain due to consecutive competitions together with seasonal darkness may further affect cognitive performance in athletes (Rosen et al., 1996).

Based on the alterations of baseline brain activity in the resting state in response to both increased light and a lack of light in the visual and sensorimotor networks, we hypothesized that sensorimotor function might be affected by ear light treatment. The aim of this study was to evaluate the effects of transcranial bright light treatment via the ear canals on cognitive performance in professional ice hockey players during the competition season. The bright light and sham interventions were organized during a very busy competition schedule during the dark time of the year (October 2011)—both components potentially resulting in overreaching. Secondly, we wanted to perform the study with one professional athletic team, since potential confusing factors like training load, competitions, and travel are virtually identical within the team. The treatments were given every morning throughout the study period and the protocol of the study was randomized, double-blind, and placebo-controlled.

## Methods

### Subjects and study protocol

Psychomotor speed tests with audio and visual warning signals and a memory test were administered to a Finnish National Ice Hockey League team (Oulun Kärpät, players' age 25 ± 5, range 17–33 years) before and after 24 days of bright light or sham treatment. The study protocol consisted of a randomized, double-blind, placebo-controlled study design. The subjects were randomized into treatment (*n* = 11, 25 ± 6 years, weight 88 ± 8 kg, and height 184 ± 6 cm) and placebo (*n* = 11, 24 ± 4 years, weight 85 ± 6 kg, and height 180 ± 7 cm) groups. The interventions were performed during seasonal darkness (October 2011) in the Oulu region (latitude 65 degrees north) when the strain on the players was also very high (10 matches during 24 days). All the subjects gave written informed consent and the investigation conforms with the principles outlined in the Declaration of Helsinki. The study was performed according to the Declaration of Helsinki, the local committee of research ethics of the Northern Ostrobothnia Hospital District approved the protocol, and all the subjects gave written informed consent. Trial has not been registered in Clinical Trials because intervention was not used to modify a health outcome.

### Bright light treatment

The brain-targeted bright light treatment or sham treatment was given transcranially via ear canals by using the VALKEE NPT 1100 bright light device (Valkee Ltd, Oulunsalo, Finland). The device was approved as a medical device in the European Union on 30 March 2010 (certificate no VTT-C-7657-01-1143-461-11). The ability of the sham device to produce light was eliminated. Otherwise the sham device worked exactly the same way as the bright light device. In order to create a real sham design, the subjects were told that effective/treating wavelengths of light are not necessarily visible and it is not possible to decide externally whether the treatment device is a sham or not. The blue-based white light was produced by light-emitting diodes (LEDs) which were attached to earplugs. In order to optimize treatment adherence, daily light treatment or sham treatment was taken at home between 8 am and noon. Each treatment session lasted 12 min which is a recommended treatment time by Valkee Ltd. The duration of treatment was based on previous observations on effectiveness of bright light therapy via ear canals on mental wellness (Timonen et al., 2012).

### Psychomotor measurements

**Figure 1:**
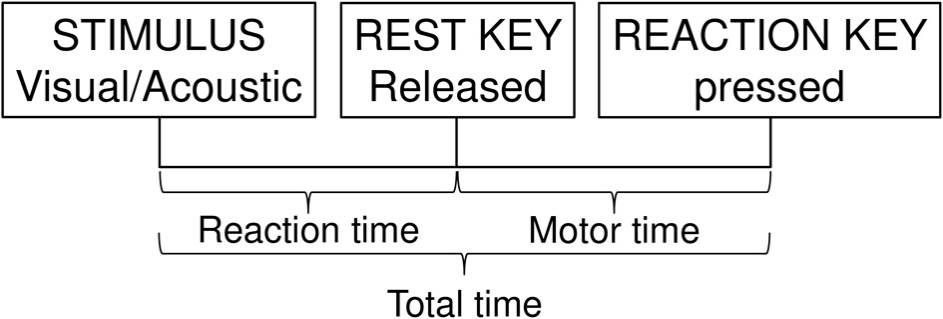
**Psychomotor performance test**. The subjects were instructed to press a reaction key when specific stimuli were presented and, having pressed the key, to return their finger immediately to the rest key. Mean reaction time (stimulus onset-reaction key pressed) and mean motor time (rest key released-reaction key pressed) were measured (the mean values for 28 warning signals from both tests). In the first part of the test the stimulus was visual (a yellow light), and in the second part, an acoustic stimulus (a beep).

An experienced psychologist conducted all the psychomotor tests (Eka Roivainen). The speed tests were administered with a Vienna Test System (Schuhfried GmbH, Moedling, Austria) and the memory test with a Cantab Test (Cambridge Cognition, Cambridge, United Kingdom) at Verve in Oulu, Finland. The tests were administered to each individual at the same time of day before and after the intervention. The testing procedure started with a simple reaction time test. The Vienna Test System's simple reaction time test assesses reaction time and motor time in response to simple visual or acoustic signals (Figure 1). The subjects were instructed to press a reaction key when specific stimuli were presented and, having pressed the key, to return their finger immediately to the rest key. Mean reaction time (stimulus onset-reaction key pressed) and mean motor time (rest key released-reaction key pressed) were measured (the mean values for 28 warning signals from both tests). In the first part of the test the stimulus was a yellow light, and in the second part, an acoustic stimulus—a beep. Reaction time and motor time were analyzed separately for both tests. The subjects were then presented a working memory test. The Cantab Spatial Span Test assesses working memory capacity. In this test white squares were shown on the computer screen. Some of the squares changed color in a variable sequence. Then the subject had to touch the same squares in the same order as displayed. The outcome measure was simply span length (2–9 boxes).

### Quality of sleep

The quality of sleep (scale: 0–10) was studied every morning at home using a visual analog scale (VAS).

### Statistics

Standard statistical methods were used to calculate means, standard deviations, and standard errors. Normal Gaussian distribution of the data was verified by the Kolmogorov-Smirnov goodness-of-fit test (*z* > 1.0). The effect of bright light treatment on each variable was assessed using analysis of variance for repeated measures with time x group and interaction (SPSS 19.0 for Windows). The analysis was done with and without an appropriate covariate (age). Age was used as a covariate since it is known to have an effect on motor and reaction times. Significant differences were further assessed using Bonferroni *post-hoc* test. Relative changes in psychomotor test values were also calculated and group-differences were tested by independent *t*-test followed by adjustment for age (ANCOVA).

## Results

### Psychomotor measurements

**Figure 2:**
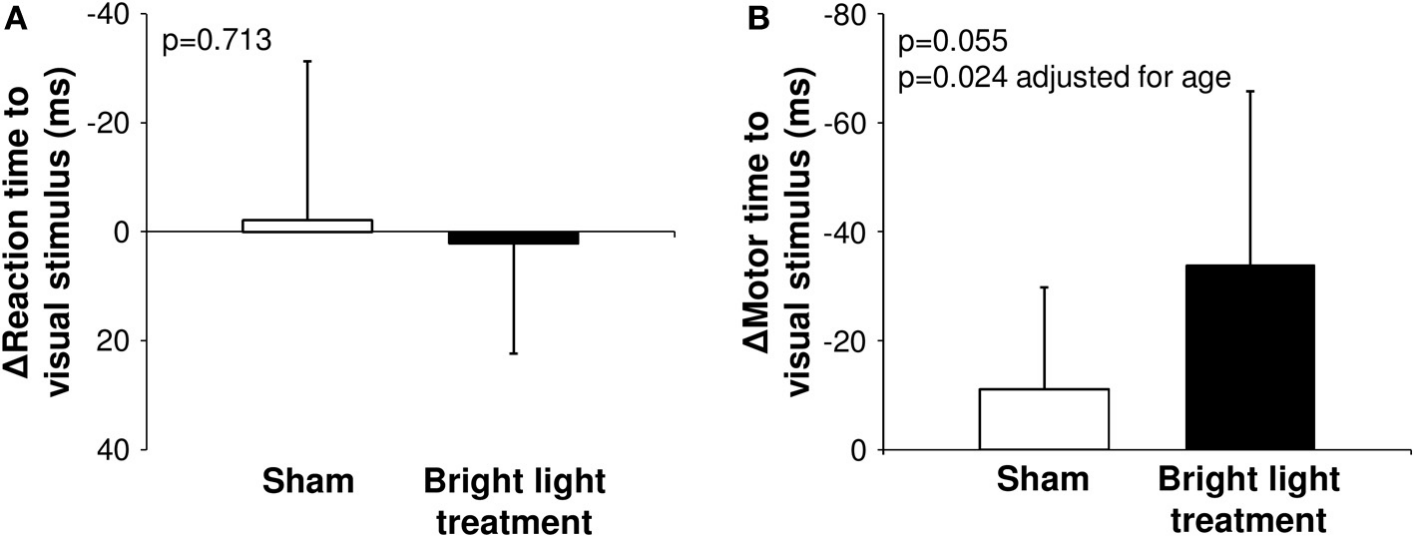
**The effects of bright light treatment via ear canals on the reaction time (A) and the motor time (B) to visual stimulus**.

The results of the psychomotor tests are shown in Table 1. Group × time interaction for motor time was *p* = 0.055 without adjustment and *p* = 0.024 after adjustment for age (Figure 2). According Bonferroni *post-hoc* test, motor time with a visual warning signal decreased in the bright light treatment group (*p* = 0.024) and did not change in the placebo group (*p* = 0.308). The relative changes in the motor time with visual stimulus were −24 ± 16% and −10 ± 15% for treatment and placebo groups, respectively (*p* = 0.044, *p* = 0.023 adjusted for age). There were no significant changes in any parameters during the audio warning test or the memory test.

**Table 1:** **Effects of bright light treatment and sham treatment on psychomotor speed *n* = 11 for both groups and group × time (treatment) interaction adjusted for age**.

	**Bright light group**		**Sham group**		**ANOVA for interaction**
	**Pre**	**Post**	**Pre**	**Post**	
**VISUAL WARNING**					
Total time, ms	256 ± 30	231 ± 36	253 ± 43	238 ± 57	*p* = 0.530
Reaction time, ms	135 ± 33	137 ± 32	136 ± 30	134 ± 45	*p* = 0.467
Motor time, ms	128 ± 43	94 ± 26^†^	121 ± 23	110 ± 32	*p* = 0.024
**AUDIO WARNING**					
Total time, ms	206 ± 37	198 ± 33	207 ± 31	201 ± 23	*p* = 0.740
Reaction time, ms	96 ± 37	98 ± 37	101 ± 30	101 ± 31	*p* = 0.747
Motor time, ms	110 ± 33	100 ± 31	109 ± 20	104 ± 23	*p* = 0.452
**MEMORY TEST**					
a.u.	7.8 ± 1.2	8.1 ± 1.2	7.8 ± 1.0	8.3 ± 0.9	*p* = 0.387

† *p < 0.05 between pre- and post-conditions*.

### Quality of sleep

The mean quality of sleep VAS scores during the treatment period were 6.3 ± 1.9 and 6.8 ± 1.9 for the treatment and sham groups, respectively (*p* = 0.582). The mean quality of sleep score during the first and last weeks were 6.3 ± 2.2 vs. 6.5 ± 2.0 and 6.9 ± 1.6 vs. 6.6 ± 1.7 for the treatment and sham groups, respectively (interaction *p* = 0.530). The quality of sleep analyzed over the whole measured period or the change in quality of sleep from the first to the last week did not correlate with any measured psychomotor variables.

## Discussion

The novel finding of the present study is that daily transcranial bright light treatment improves motor time with a visual warning signal in professional ice-hockey players measured in laboratory conditions. The finding is in line with altered resting state activity in both visual and sensorimotor networks, shown to occur during immediate light treatment and during repetitively occurring darkness-related SAD (Starck et al., 2012; Abou Elseoud et al., 2014). The daily bright light treatment was administered during the darkest time of the year (October 30; sunrise 8:02 am and sunset 16:00 pm with 55 min of twilight) and during the busy competition season (10 matches during 24 days), both potentially resulting in psychomotor slowness as an early marker of overreaching. The memory test expressing executive cognitive performance did not change in the present study, which is in line with a previous study performed with blue-enriched conventional bright light treatment (Chellappa et al., 2011). Interestingly, the auditory cued reaction speed was not altered after light treatment, matching perfectly the fMRI results showing no reactivity to either immediate light treatment or darkness SAD (Starck et al., 2012; Abou Elseoud et al., 2014).

### Potential physiological mechanisms

It is widely accepted that conventional bright light treatment improves acutely cognitive performance, and particularly motor and reaction times, in healthy populations (Campbell and Dawson, 1990; Badia et al., 1991; Daurat et al., 1993; Cajochen et al., 2000; Phipps-Nelson et al., 2003; Lockley et al., 2006; Ruger et al., 2006; Chellappa et al., 2011). Despite very well documented effects of bright light exposure on performance, we were not able to find any studies concerning the effects of bright light treatment on cognitive performance in athletes. In the present study, we investigated the effects of 24 days of bright light treatment on cognitive performance in professional athletes. The bright light was administered transcranially toward the brain via the ear canals. The ear canals offer the closest access to the brain via the transparent tympanic membrane and temporal bone. Importantly, the ear canal avoids two of the most light-absorbing tissues in the human body, namely the skin and blood. However, ambient light is able to penetrate the mammalian skull (Ganong et al., 1963), and according to recent studies, brain tissue seems to be sensitive to direct light and the lack of it during the dark time of the year (Kojima et al., 2011; Starck et al., 2012; Timonen et al., 2012).

Improvement in psychomotor performance after acute bright light exposure has been shown to be moderately associated with melatonin suppression (Chellappa et al., 2011). However, conventional bright light exposure during the day still improves cognitive performance when melatonin is undetectable, suggesting that improved performance is not only mediated via the melatonin suppression pathway but also via other mechanisms (Phipps-Nelson et al., 2003; Ruger et al., 2005). In our recent study, acute transcranial bright light exposure did not affect melatonin levels measured during day and night time hours (Jurvelin et al., 2012). Therefore, it is not supposed that melatonin suppression could explain the present finding, but that other potential non-visual pathways are involved.

The cascade, which converts photic energy into neural responses, is called phototransduction. Recent studies show that potentially photosensitive opsins are not only expressed in the mammalian retina, but also widely in the human brain (Kojima et al., 2011; Nissilä et al., 2012). At least, encephalopsin (OPN3), melanopsin (OPN4), and neuropsin (OPN5) are expressed in mice and human brains at mRNA and/or protein levels (Kojima et al., 2011; Hawrylycz et al., 2012; Nissilä et al., 2012). It is interesting that encephalopsin and melanopsin are expressed in high quantities in the cerebral cortex, hypothalamus, and cerebellum, which is known to modulate e.g., motor function and attention (Stoodley and Schmahmann, 2010). A significant amount of light penetrates the skull bone and reach the brain (Ganong et al., 1963), and non-visual phototransduction via the opsins pathway are one plausible explanation for the present findings of improved alertness and cognitive performance in professional athletes. However, more studies on the existence and function of opsins in the human brain are warranted.

The most advanced bright light exposure studies are using fMRI techniques to study in more detail the effects of bright light treatment on various brain areas (Vandewalle et al., 2006, 2007a,b, 2010, 2011). In these studies the effects of bright light exposure have been shown to be dependent on duration, photon density, and wavelength (Vandewalle et al., 2009). Blue-enriched light is the most powerful wavelength of light resulting in activation of various brain areas, including e.g., the brainstem (Vandewalle et al., 2007b), the hypothalamus (Perrin et al., 2004), and the thalamus (Vandewalle et al., 2006, 2007a,b), which relay sensory and motor signals to the cerebral cortex in humans (Portas et al., 1998). Blue-enriched light was also used in the present study. Starck et al. recently showed acutely increased functional connectivity in lateral visual and sensorimotor networks in a transcranial bright light group compared with a sham group by using blood oxygen level dependent (BOLD) fMRI techniques. These findings were detected during blinded, sham-controlled, 8-min transcranial bright light exposure of the brain via the ear canals inside a MRI scanner (Starck et al., 2012). The interesting fact is that the changes in fluctuation that occurred in the visual and motor cortex are in accordance with the findings of the present study. This preliminary and novel finding by Starck et al. supports the present study, where particularly the motor component of cognitive performance was improved in response to the visual stimulus. It is also possible that the results of the present study highlight differences in visual vs. auditory cue processing rather than the motor reaction time. However, more studies on these potential differences are warranted.

### Limitation

The number of subjects in the present study was small which limits the statistical power of the results. However, we wanted to perform the study with one professional athletic team, since training load, competitions, and travel are virtually identical within the team. Our principle finding on group-differences in absolute changes in the motor time to visual stimulus was statistically significant only when adjusted for age, although evident tendency was observed without adjustments. We considered adjustment for age relevant because of the previous findings on declining motor speed with aging and significant correlation between the change of motor time to visual stimulus and age (*r* = 0.62, *p* = 0.044), despite age did not differ between the treatment and sham groups. However, evident group-differences were observed in the relative changes in the motor time to visual stimulus also without adjustments for age, supporting the present conclusions. The fMRI measurements would have been important information on physiological mechanisms of light in pre and post-condition. However, this was not possible due to the extreme busy time schedule of the athletes. Some improvements could be partly explained with learning and/or placebo effects. We told the subjects that effective/treating wavelengths of light are not necessarily visible and it is not possible to decide externally whether the treatment device is a sham or not. However, the subjects knew that this study is bright light study and therefore some subjects may know their group membership.

## Conclusion

Transcranial bright light treatment via the ear canals may improves motor time with a visual warning signal in elite athletes, suggesting that brain tissue is responsive to direct light exposure.

### Conflict of interest statement

Mikko P.Tulppo, Eka Roivainen, Arto J. Hautala, Antti M. Kiviniemi, and Vesa J. Kiviniemi have no conflicts of interests. Heidi Jurvelin works for Valkee Ltd, Juuso Nissilä is the company founder and a shareholder, and Timo Takala is a minor shareholder.

## References

[B1] Abou Elseoud A. Nissila J. Liettu A. Remes J. Jokelainen J. Takala T. (2014). Altered resting-state activity in seasonal affective disorder. Hum. Brain Mapp. 35, 161–172 10.1002/hbm.22164 22987670 PMC6869738

[B2] Badia P. Myers B. Boecker M. Culpepper J. Harsh J. R. (1991). Bright light effects on body temperature, alertness, EEG and behavior. Physiol. Behav. 50, 583–588 10.1016/0031-9384(91)90549-4 1801013

[B3] Blackshaw S. Snyder S. H. (1999). Encephalopsin: a novel mammalian extraretinal opsin discretely localized in the brain. J. Neurosci. 19, 3681–3690 10234000 10.1523/JNEUROSCI.19-10-03681.1999 PMC6782724

[B4] Cajochen C. Zeitzer J. M. Czeisler C. A. Dijk D. J. (2000). Dose-response relationship for light intensity and ocular and electroencephalographic correlates of human alertness. Behav. Brain Res. 115, 75–83 10.1016/S0166-4328(00)00236-9 10996410

[B5] Campbell S. S. Dawson D. (1990). Enhancement of nighttime alertness and performance with bright ambient light. Physiol. Behav. 48, 317–320 10.1016/0031-9384(90)90320-4 2255738

[B6] Chellappa S. L. Steiner R. Blattner P. Oelhafen P. Gotz T. Cajochen C. (2011). Non-visual effects of light on melatonin, alertness and cognitive performance: can blue-enriched light keep us alert? PLoS ONE 6:e16429 10.1371/journal.pone.0016429 21298068 PMC3027693

[B7] Daurat A. Aguirre A. Foret J. Gonnet P. Keromes A. Benoit O. (1993). Bright light affects alertness and performance rhythms during a 24-h constant routine. Physiol. Behav. 53, 929–936 10.1016/0031-9384(93)90271-G 8511209

[B8] Ganong W. F. Shepherd M. D. Wall J. R. Van Brunt E. E. Clegg M. T. (1963). Penetration of light into the brain of mammals. Endocrinology 72, 962–963 10.1210/endo-72-6-962 13946401

[B9] Hawrylycz M. J. Lein E. S. Guillozet-Bongaarts A. L. Shen E. H. Ng L. Miller J. A. (2012). An anatomically comprehensive atlas of the adult human brain transcriptome. Nature 489, 391–399 10.1038/nature11405 22996553 PMC4243026

[B10] Hynynen E. Uusitalo A. Konttinen N. Rusko H. (2008). Cardiac autonomic responses to standing up and cognitive task in overtrained athletes. Int. J. Sports Med. 29, 552–558 10.1055/s-2007-989286 18050058

[B11] Jurvelin H. Kallio L. Kiviniemi V. Leppäluoto J. Nissilä J. Saarela S. (2012). Does light have psychophysiological non-image-forming (NIF) -effects outside of retinohypothalamic tract (RHT)? Int. J. Psychiatry Clin. Pract. 16(suppl. 1), 32

[B12] Kojima D. Mori S. Torii M. Wada A. Morishita R. Fukada Y. (2011). UV-sensitive photoreceptor protein OPN5 in humans and mice. PLoS ONE 6:e26388 10.1371/journal.pone.0026388 22043319 PMC3197025

[B13] Lockley S. W. Evans E. E. Scheer F. A. Brainard G. C. Czeisler C. A. Aeschbach D. (2006). Short-wavelength sensitivity for the direct effects of light on alertness, vigilance, and the waking electroencephalogram in humans. Sleep 29, 161–168 16494083

[B14] Nederhof E. Lemmink K. A. Visscher C. Meeusen R. Mulder T. (2006). Psychomotor speed: possibly a new marker for overtraining syndrome. Sports Med. 36, 817–828 10.2165/00007256-200636100-00001 17004845

[B15] Nederhof E. Lemmink K. Zwerver J. Mulder T. (2007). The effect of high load training on psychomotor speed. Int. J. Sports Med. 28, 595–601 10.1055/s-2007-964852 17373595

[B16] Nissilä J. Mänttäri S. Särkioja T. Tuominen H. Takala T. Timonen M. (2012). Encephalopsin (OPN3) protein abundance in the adult mouse brain. J. Comp. Physiol. A 198, 833–839 10.1007/s00359-012-0754-x 22991144 PMC3478508

[B17] Perrin F. Peigneux P. Fuchs S. Verhaeghe S. Laureys S. Middleton B. (2004). Nonvisual responses to light exposure in the human brain during the circadian night. Curr. Biol. 14, 1842–1846 10.1016/j.cub.2004.09.082 15498492

[B18] Phipps-Nelson J. Redman J. R. Dijk D. J. Rajaratnam S. M. (2003). Daytime exposure to bright light, as compared to dim light, decreases sleepiness and improves psychomotor vigilance performance. Sleep 26, 695–700 14572122 10.1093/sleep/26.6.695

[B19] Portas C. M. Rees G. Howseman A. M. Josephs O. Turner R. Frith C. D. (1998). A specific role for the thalamus in mediating the interaction of attention and arousal in humans. J. Neurosci. 18, 8979–8989 9787003 10.1523/JNEUROSCI.18-21-08979.1998 PMC6793555

[B20] Rosen L. W. Shafer C. L. Smokler C. Carrier D. McKeag D. B. (1996). Seasonal mood disturbances in collegiate hockey players. J. Athl. Train. 31, 225–228 16558403 PMC1318508

[B21] Ruger M. Gordijn M. C. Beersma D. G. De Vries B. Daan S. (2005). Weak relationships between suppression of melatonin and suppression of sleepiness/fatigue in response to light exposure. J. Sleep Res. 14, 221–227 10.1111/j.1365-2869.2005.00452.x 16120096

[B22] Ruger M. Gordijn M. C. Beersma D. G. De Vries B. Daan S. (2006). Time-of-day-dependent effects of bright light exposure on human psychophysiology: comparison of daytime and nighttime exposure. Am. J. Physiol. Regul. Integr. Comp. Physiol. 290, R1413–R1420 10.1152/ajpregu.00121.2005 16373441

[B23] Starck T. Nissilä J. Aunio A. Abou-Elseoud A. Remes J. Nikkinen J. (2012). Stimulating brain tissue with bright light alters functional connectivity in brain at the resting state. Word J. Neurosci. 2, 81–90 10.4236/wjns.2012.22012

[B24] Stoodley C. J. Schmahmann J. D. (2010). Evidence for topographic organization in the cerebellum of motor control versus cognitive and affective processing. Cortex 46, 831–844 10.1016/j.cortex.2009.11.008 20152963 PMC2873095

[B25] Timonen M. Nissila J. Liettu A. Jokelainen J. Jurvelin H. Aunio A. (2012). Can transcranial brain-targeted bright light treatment via ear canals be effective in relieving symptoms in seasonal affective disorder? A pilot study. Med. Hypotheses 78, 511–515 10.1016/j.mehy.2012.01.019 22296809

[B26] Vandewalle G. Balteau E. Phillips C. Degueldre C. Moreau V. Sterpenich V. (2006). Daytime light exposure dynamically enhances brain responses. Curr. Biol. 16, 1616–1621 10.1016/j.cub.2006.06.031 16920622

[B27] Vandewalle G. Gais S. Schabus M. Balteau E. Carrier J. Darsaud A. (2007a). Wavelength-dependent modulation of brain responses to a working memory task by daytime light exposure. Cereb. Cortex 17, 2788–2795 10.1093/cercor/bhm007 17404390

[B28] Vandewalle G. Hebert M. Beaulieu C. Richard L. Daneault V. Garon M. L. (2011). Abnormal hypothalamic response to light in seasonal affective disorder. Biol. Psychiatry 70, 954–961 10.1016/j.biopsych.2011.06.022 21820647 PMC5323254

[B29] Vandewalle G. Maquet P. Dijk D. J. (2009). Light as a modulator of cognitive brain function. Trends Cogn. Sci. 13, 429–438 10.1016/j.tics.2009.07.004 19748817

[B30] Vandewalle G. Schmidt C. Albouy G. Sterpenich V. Darsaud A. Rauchs G. (2007b). Brain responses to violet, blue, and green monochromatic light exposures in humans: prominent role of blue light and the brainstem. PLoS ONE 2:e1247 10.1371/journal.pone.0001247 18043754 PMC2082413

[B31] Vandewalle G. Schwartz S. Grandjean D. Wuillaume C. Balteau E. Degueldre C. (2010). Spectral quality of light modulates emotional brain responses in humans. Proc. Natl. Acad. Sci. U.S.A. 107, 19549–19554 10.1073/pnas.1010180107 20974959 PMC2984196

